# Responding to the Pandemic of Falsified Medicines

**DOI:** 10.4269/ajtmh.14-0393

**Published:** 2015-06-03

**Authors:** Gaurvika M. L. Nayyar, Amir Attaran, John P. Clark, M. Julia Culzoni, Facundo M. Fernandez, James E. Herrington, Megan Kendall, Paul N. Newton, Joel G. Breman

**Affiliations:** Johns Hopkins Bloomberg School of Public Health and Johns Hopkins Carey Business School, Baltimore, Maryland; Population Health and Global Development Policy, University of Ottawa, Ottawa, Ontario, Canada; Pfizer Global Security, Pfizer Pharmaceuticals, New York, New York; School of Chemistry and Biochemistry, Georgia Institute of Technology, Atlanta, Georgia; Gillings Global Gateway, Gillings School of Global Public Health, University of North Carolina at Chapel Hill, Chapel Hill, North Carolina; School of Law, University of Ottawa, Ottawa, Ontario Canada; Lao-Oxford-Mahosot Hospital-Wellcome Trust Research Unit, Microbiology Laboratory, Mahosot Hospital, Vientiane, Lao PDR; Centre for Tropical Medicine, Churchill Hospital, Nuffield Department of Medicine, University of Oxford, Oxford, United Kingdom; Fogarty International Center, National Institutes of Health, Bethesda, Maryland

## Abstract

Over the past decade, the number of countries reporting falsified (fake, spurious/falsely labeled/counterfeit) medicines and the types and quantities of fraudulent drugs being distributed have increased greatly. The obstacles in combating falsified pharmaceuticals include 1) lack of consensus on definitions, 2) paucity of reliable and scalable technology to detect fakes before they reach patients, 3) poor global and national leadership and accountability systems for combating this scourge, and 4) deficient manufacturing and regulatory challenges, especially in China and India where fake products often originate. The major needs to improve the quality of the world's medicines fall into three main areas: 1) research to develop and compare accurate and affordable tools to identify high-quality drugs at all levels of distribution; 2) an international convention and national legislation to facilitate production and utilization of high-quality drugs and protect all countries from the criminal and the negligent who make, distribute, and sell life-threatening products; and 3) a highly qualified, well-supported international science and public health organization that will establish standards, drug-quality surveillance, and training programs like the U.S. Food and Drug Administration. Such leadership would give authoritative guidance for countries in cooperation with national medical regulatory agencies, pharmaceutical companies, and international agencies, all of which have an urgent interest and investment in ensuring that patients throughout the world have access to good quality medicines. The organization would also advocate strongly for including targets for achieving good quality medicines in the United Nations Millennium Development Goals and Sustainable Development Goals.

## The Problem

Malaria is a devastating illness, particularly to young children and pregnant women in tropical countries. A recent review reported that the active pharmaceutical ingredient (API) was absent in over one-third of close to 4,000 antimalarial drug samples tested from pharmacies in seven southeast Asian and 21 sub-Saharan African countries^1^; over 40% of the alleged artemisinin-containing drugs were falsified, outright fakes. A wide variety of falsified brand name and generic medicines and even falsified raw ingredients for several essential pharmaceuticals have been found in rich and poor countries.^2^–^7^ Such drugs are often used for acutely ill patients, many of whom would die or suffer prolonged illness without proper treatment. In addition to patients' loss of confidence in the health-care delivery system, microbial resistance to the drug may develop and spread if medicines contain subtherapeutic doses or no API. The increasing global scientific and public awareness and epidemic proportions of the spreading problem are reflected in the number of articles on “fake drugs” cited in PubMed: 27 papers from 1966 to 1999, 56 papers from 2000 to 2004, 122 papers from 2005 to 2009, and 294 papers from 2010 to 2015 (February).

**Figure 1.**
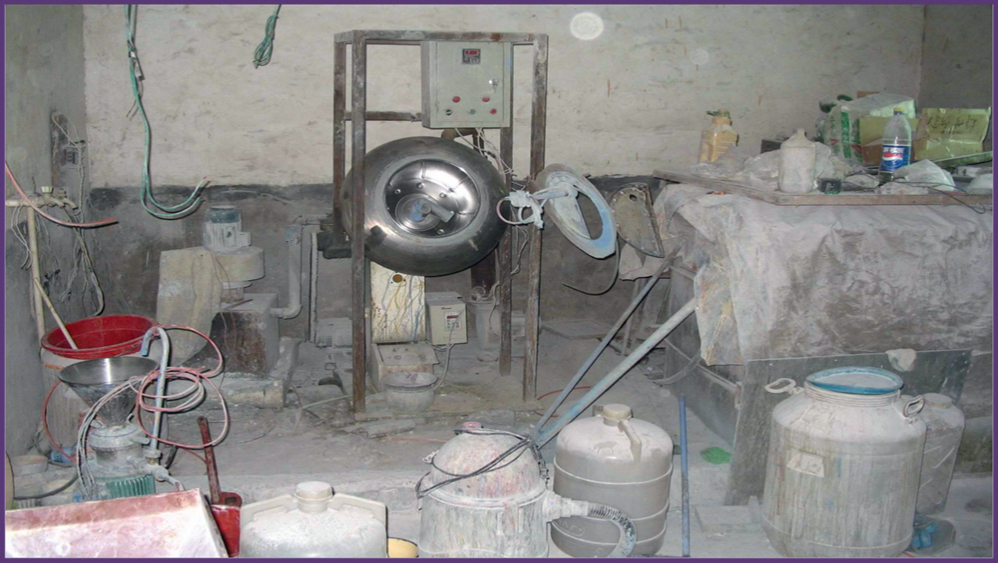
Facility producing fraudulent drugs.

Until recently, there were a paucity of reports from pharmaceutical companies on the type and quantity of drugs that were fraudulently compounded or transferred by criminals. Data are emerging from the Pharmaceutical Security Institute (PSI), a not-for-profit membership organization of pharmaceutical security directors, indicating that a large number of companies, products, and countries are targeted.^8^ For instance, since 2008, Pfizer Pharmaceuticals (Pfizer Global Security, New York, NY) has identified a rapidly increasing number of falsified products, countries reporting falsified drugs, and breaches of the legitimate supply chain national entry points (Table 1); the increases have been from 40% to over 100%. Of Pfizer products, those for erectile dysfunction are most frequently falsified^9^; other such products target patients with Alzheimer's disease, cancer, high cholesterol, hypertension, malaria, and anxiety disorder. Facilities where fake drugs were made or compounded were discovered with moldy walls, dirty equipment, and infested with rodents and insects (Figure 1). Falsifiers have created products that are visually indistinguishable from the genuine product, clearly demonstrating criminal intent to deceive. This increasingly recognized problem on virtually all continents is a pandemic defined as “an epidemic occurring over a very wide area, crossing international boundaries, and usually affecting a large number of people.”^10^

## Definitions

Despite increasing awareness of the fraudulent drug epidemic, efforts to quantify and stop this peril have been stymied by multiple obstacles, not the least of which is agreement on definitions.^4^,^11^,^12^ Poor quality drugs include substandard/spurious/falsely labeled/falsified/counterfeit (SSFFC) medical products.^4^ Falsified (also commonly called fake or counterfeit products are *intentionally and fraudulently produced* and contain no API, the incorrect dose of the API, or the incorrect API. Substandard medicines are caused by *unintentional or negligent errors of manufacturing* or by *degradation* after manufacturing resulting in insufficient API, poor dissolution properties, or degradation products. The nomenclature used by the World Health Organization (WHO), the World Trade Organization, the United Nations (U.N.) Office on Drugs and Crime, INTERPOL, and others can be confusing; hence, we are using terms agreed upon by WHO.^4^

There are also properly manufactured medicines that are unlawful for reasons apart from their quality. These can be unregistered with company branded or generic medicines that, for reasons of theft or accidental or intentional diversion, do not have the legally required marketing authorization of the country's regulators to be imported or sold there, and medicines that infringe the trademark of a legal product. Relatively little is known about medicines that have expired and are repackaged with a new date; these topics along with diverted products are beyond the realm of this article. This article focuses mainly on medical and public health considerations of falsified medicines that are particularly widespread in low- and middle-income countries.^13^–^15^ Although there are increasing reports of detection of a variety of fake drugs from around the world, paradoxically, there are virtually no reports from middle- or high-income countries quantifying the state of poor quality medicines, only anecdotal case citations.

## Overview of Control Challenges

Governments have been hampered by a confusing array of expensive detection technologies. Few functional national regulatory authorities exist in low-income nations that lack trained staff and suitably equipped laboratories to test drug quality centrally or in peripheral pharmacies or markets.^13^,^14^ Furthermore, the variability or absence of national and international criminal statutes, lack of an international agreement against trafficking of poor quality medicines, and inadequate punishments for convicted offenders reflect the weak legal framework for confronting drug fraud. One of the biggest obstacles in provision of quality-assured pharmaceuticals is the lack of effective manufacturing, regulatory, and quality processing in India and China. In 2013, 10 global public health agencies including providers, foundations, and research institutions contributed to developing an advocacy campaign to address falsified medicines, particularly in China. This campaign called *Fight the Fakes* is a step toward raising awareness about the problem, but legal action has to follow along with more public and political awareness.^15^

The U.S. Institute of Medicine (IOM) has published a report “Countering the Problem of Falsified and Substandard Drugs.”^16^ The IOM recommendations to “stem the global trade” in such products are laudable in advising that the U.S. Food and Drug Administration (FDA), the National Institute of Standards and Technology, and other U.S. and international pharmaceutical and financing agencies be more actively involved in setting standards and financing improvements; yet this report *falls far short of making a strong call for standardized, agreed-upon quality assessment technologies; an international law convention; and a more activist, internationally recognized lead organization, all three of which are essential for stopping the many health threats of fake drugs*. Global leadership to date has devolved in parts to the WHO, the U.N. Office on Drugs and Crime, and INTERPOL, each with diverse missions, responsibilities, limited authorities, and their own collaborations, funding networks, cultures, and languages.^17^ No organization is leading assertively. Of the three areas listed, an international convention and improved national regulations are likely to have the most enduring value in concert with effective leadership and other innovations. The focus of all actions tied to drug quality must be on public and individual health, and strengthening national capacities to improve the health of their citizens.

## Policy Proposals

### Detection methods and technology.

A major hindrance to understanding the types, names, extent, and amount of poor quality drugs nationally and globally has been 1) the lack of agreed-upon field survey approaches^18^ and 2) available low-cost tools to detect and classify bad drugs quickly at points of entry into countries, at public and private pharmacies, and in health units. In 2014, the WHO published draft guidelines for surveys of medicine quality that are currently being revised.^19^

Two or more levels of drug quality tests exist: 1) methods useable in the field that are quick, inexpensive, and easy to use and teach; these methods are targeted mainly to examine packaging and detect drug contents and 2) technologies requiring a laboratory equipped for exhaustive chemical analysis. These approaches are summarized in Table 2, deriving from the IOM report^16^ and a recent analysis by Green and others^20^ from the Centers for Disease Control and Prevention reference laboratory. Within each method there are numerous tools and prototypes being used and new ones tested. Current technologies for field use rely on visual packaging inspection, lot number reporting via mobile phones, thin-layer chromatography, colorimetric tests, and simplified spectroscopic methods. Gas and liquid chromatography and mass spectrometry are some of the more advanced and complex techniques for investigating drug quality in central laboratories. Qualitative or semiquantitative tests for an API are not substitutes for proper manufacturing control, dissolution studies, pharmacokinetics equivalence, and supply chain integrity. A very promising recent development has been the U.S. Pharmacopeia Promoting the Quality of Medicines (PQM) program in several African, Asian, and Latin American countries using the “Minilab” (Global Pharma Health Fund e.V., Giessen, Germany).^21^–^23^ This training program supported by the U.S. Agency for International Development (USAID) and the President's Malaria Initiative (PMI) has trained several hundred persons in rapid chemical analysis of drugs taken from public and private pharmacy stocks.^23^ A major reference training center has recently been opened in Accra, Ghana, with USAID and U.S. Pharmacopeia support as a referral testing and regional training center. Important also is the development of the counterfeit detection (CD)-3 (US Food and Drug Administration, Forensic Chemistry Center, Cincinnati, OH), a promising handheld electronic device for peripheral use that detects fake packaging at point of sale with images and videos of the suspect samples.^24^,^25^ The FDA, Skoll Foundation, and other partners are supporting expansion of testing and use of this device. We recommend that a precertification of essential diagnostics, drugs, and vaccines should be required for specific regional and global control, elimination, and eradication programs and campaigns. More information is needed to confirm that precertification of products is occurring for the PMI and the Global Fund to Fight AIDS, Tuberculosis and Malaria, and products purchased by U.N. International Children's Emergency Fund (UNICEF) and WHO. Essential drugs designated by WHO should also be targeted for special vigilance by quality assurance mechanisms.

No independent agency has inventoried and performed comparative quality assessments of these packaging and drug-testing devices and made recommendations to countries for their use. Objective comparisons are needed of the diversity of field methods in terms of accuracy, reliability, costs of equipment and supplies, level of training needed, ease of use, spare part availability, and maintenance requirements. Simplified standard survey protocols and methods for sampling drugs at country entry points (seaports, airports, and roads); at major pharmacy depots; in health units (public and private hospitals and clinics); and at more peripheral distribution sites (district and village pharmacies and individual vendors) are also needed.^19^ Low-cost, portable detection tools would empower pharmaceutical inspectors in numerous countries that have oversight of the medicine supply. Results would be available promptly rather than delayed when samples are sent to national or international laboratories as occurs now; lamentably, intervals of several years have occurred from the time specimens were collected to the time the results were available to those needing to take action.^1^ Ideally, central reference laboratories vetted by WHO, FDA, or another agency would back up spot checks and random sampling of pharmaceuticals at the periphery.

### Good quality medicines by law.

Falsified medicines are ultimately a problem that impacts public health. The solution needs to reflect various incentives, either via financial gain, avoidance of punishment or both. A multi-sectorial effort is essential for taking into account how this illegal market is interwoven with world trade agreements, business models, and associated legal ramifications. Globalization has enlarged the international trade in medicines. For example, India exports over US$15.5 billion in pharmaceuticals, which are among their most important exports.^26^ As of 2011, 40% of drugs and 80% of APIs for drugs in the United States are imported from foreign countries.^27^ An international law convention against substandard and falsified medicines would address both regulatory and criminal international governance challenges simultaneously through technical, legal, and financial mechanisms.

### How would the convention work and what national benefits would it bring?

A convention would provide four legal underpinnings that do not exist, that together would advance patient safety and access to quality medicines.^12^ First, a convention would define the various sorts of wrongful medicines accurately and thereby avoid misunderstandings caused by today's problematic or vague terminology (e.g., where countries seized good quality generic medicines as “counterfeit”). Second, a convention would promote the requirement that signatory countries enact national laws to designate wrongful acts—such as the intentional manufacture, trafficking, or selling of falsified medicines—as criminal offences, with attendant obligations to alert health-care workers and to prosecute or extradite the offenders to justice promptly. Third, a convention would provide the legal and institutional framework for participating countries to agree, implement, and evolve convergent standards of medicine regulation, so as to reduce poor quality medicines in international trade. Fourth, and for lower income countries particularly, a convention would contain mechanisms for financial and technical assistance, and, to join local and regional networks. These actions would help build national and regional medicine regulatory authorities (MRAs) to a point where patients' access to quality medicines is protected.

Some have said that establishing recommended codes of practice that are nonbinding (soft law) are better than international norms and regulations that are binding (hard law).^28^ We disagree with soft law in regard to controlling the current fake-drug pandemic. There are precedents for using international law in this way. A 1929 treaty that internationally criminalizes counterfeit banknotes provides an analogy for falsified medicines. In the health field, there are treaties specifically addressing the illicit traffic of certain narcotic drugs and treaties to prevent harm—particularly, the Framework Convention on Tobacco Control and its associated protocols to stanch illicit trade. That convention has brought over US$250 million new funding to global tobacco-control efforts, demonstrating that international law need not compete for resources, but can increase them.^29^ The U.N. Office on Drugs and Crime has been developing “Draft Model Legislative Provisions on Fraudulent Medical Products” for several years but there has been no agreement on final text; the focus appears to be on criminal and judicial issues.

### Challenges ahead.

Information is accruing that large quantities of falsified drugs are being manufactured in Asian countries. China and India are two of the largest producers of good quality drugs and vaccines, many of which are purchased or funded by the USAID; UNICEF; Global Fund to Fight AIDS, Tuberculosis and Malaria; WHO; and other organizations, charities, and national agencies for global disease control and eradication programs. However, according to the World Customs Organization, in 2006, 54% and 21% of unlawful drugs of all sorts confiscated worldwide were manufactured in India and China, respectively.^30^ The circuitous travel itineraries of fake medicines have been traced across continents, such that the unsuspecting recipient countries assume a bona fide origin. A particularly heinous example is that of multiples instances of the production, marketing, and international travel of falsified bevacizumab (Avastin^®^), a cancer medicine; the fake drug closely matched the appearance of the real medicine, but tests indicated salt, starch, and various cleaning solvents instead of the active ingredient with resulting endophthalmitis.^31^

The Internet has opened up an unregulated opportunity for criminals to promote and sell fake drugs to unsuspecting vulnerable populations, often the aged and others seeking convenience and low cost. A recent survey of over 10,000 online pharmacies found that 96% operated outside legal regulations and a large percentage closed operations within 3 years of operation.^32^,^33^ The FDA and other organizations participate with INTERPOL in annual international actions (Operation Pangea) to shutdown illegal pharmacy websites selling potentially counterfeit and illegal medical products. More than 18,000 such illegal websites were closed during one week in 2012 with seizure of US$10.5 million of pharmaceuticals worldwide.^34^

### Leadership, collaboration, and national strengthening.

Arguably, the major obstacle to solving the problem of poor quality medicines has been the lack of a clearly identified lead organization with a plan of action developed in concert with countries, pharmaceutical companies (multinational corporations and innovator/biotechnology enterprises), and national and international agencies—and a sense of urgency to implement the plan with resources and partners—including pharmaceutical companies in low-income countries. WHO has estimated that 30% of countries have inadequate medicines regulation authorities (MRAs) or none at all. Moreover, WHO has found that 90% of African MRAs lack the capacity to undertake medicine regulatory functions and therefore cannot guarantee the quality, efficacy, and safety of medicines,^35^,^36^ The New Partnership for Africa's Development has found that there is either limited or declining government funding for MRAs in the East African Community Partner States.^37^

Many have looked to WHO for this leadership, given its successful implementation of the public health treaty on tobacco control. However, some argue that the U.N. system, including the WHO, is poorly suited to be in a leadership role because of sparse technical expertise in products, manufacturing, and quality systems. U.N. agencies are beholden to member states and cannot regulate or enforce anything easily, especially, in India and China. In this regard, WHO could serve the role of a partner rather than a leader. The recently revitalized Rapid Alert program at WHO has begun to “track and trace” poor quality drugs as reported voluntarily by member countries.^38^ Rapid Alert notices are published periodically by WHO indicating the fake drug type, lot number, quantity of product, and place detected. Strong action by countries can stem the tide as shown in Rwanda^39^ and Cambodia,^40^ although unique situations and major multi-sectorial engagements exist in these countries. One solution is creation of regional harmonization networks, addressing some elements of drug registration tied to regional economic communities; the African Medicines Registration Harmonization Initiative is one example of such a network. The U.N. Office on Drugs and Crime (UNODC) has also made recent attempts at facilitating international cooperation against falsified medicines. One proposal has been a trilateral coalition of the UNODC, WHO, and Interpol.^17^ Still, active and transparent support from the FDA, drug companies, individual countries, and other partners will be needed; the FDA may be the most qualified as a leadership organization based on their technical expertise and global influence. Mechanisms for training technical staff, regulating products, improving manufacturing practices, and stopping criminal production are needed to assure a good supply of medicines. Given that the problem of substandard and falsified medicines should be approached primarily from a public health and equity perspective, it is important that the negotiations on the way forward be led by the Ministries of Health along with the Ministers of Finance and Trade, while respecting legitimate intellectual property rights. Could and should WHO be the lead organization in curbing the spreading epidemic of falsified pharmaceuticals? WHO's ability to take more assertive action is strengthened by the revised international health regulations. WHO's director general can convene emergency committees in response to public health emergencies as has been done recently for the influenza A (H1N1) pandemic in 2009, the middle east respiratory syndrome (MERS-CoV) in 2013, the polio crises in 2014, and the Ebola epidemic in 2014–2015. Illicit drug trafficking is an emergency. The Drug Quality and Security Act, signed into law by President Obama in 2013, outlines steps to build an electronic system to identify and trace certain prescription drugs in the United States. The results of this system are awaited.

Finally, the Millennium Development Goals (MDGs), under revision, should include measurable objectives for good quality drugs. This will encourage national establishment of baseline status and achievable targets, particularly for essential drugs. Establishment of MDG targets and Sustainable Development Goals (SDGs) will help greatly to solve the poor quality drug epidemic by application of available technology and good pharmaceutical vigilance and governance.^41^ One incentive that would transform the current system is applying a “universal quality standard” to drug products. For example, if India allows a substandard manufacturer to sell products in Africa, the FDA could ban import of products from India. Although difficult to develop and implement, a combination of incentives and penalties driven at the political and economic levels is needed.

## Conclusions

The major urgent needs to improve the quality of the world's medicines fall into three main areas: 1) research to develop and compare the most accurate and affordable tools to identify high-quality drugs at point of sale and deployment of the best methods; 2) an international convention and national legislation to facilitate production and use of high-quality drugs and protect all countries from the criminal and the negligent who make, distribute, and sell life-threatening products; 3) designation of a highly qualified, well-supported international organization, possibly the FDA or WHO, that will establish standards, training programs, drug quality surveillance, and authoritative guidance for countries in cooperation with national medical regulatory agencies, pharmaceutical companies, and international agencies, all of which have an urgent interest and investment in ensuring that patients throughout the world have access to good quality medicines. The organization would also advocate strongly for including targets for achieving good quality medicines in the MDGs and SDGs including certification of pharmaceutical products entering countries that request such services, and participate in global disease control and elimination programs.

## Figures and Tables

**Table 1:** Falsified drugs and drugs entering the supply chain illegitimately

	2008	2014
Countries reporting falsified drugs	75	107
Falsified products	29	69
Breaches of legitimate supply chain entry		
Medicines	8	26
Countries	25	60

Countries and products reported by Pfizer global security.

**Table 2:** Technologies for testing poor quality medicines

Tests useable in field	Detects
Visual inspection of package and product	Wrong package, color, size, shape, and spelling
Alternative light sources	Wrong color of ink, holograms, packaging, excipients in pills (wavelengths in the visible (350–700 nm) and nonvisible (> 700 nm) electromagnetic spectrum
Colorimetry	Wrong kind and/or amounts of API
Simplified disintegration tests	Disintegration, as marker for bioavailability
“Minilab”	Compendium of thin-layer chromatography and simplified disintegration tests
Raman spectroscopy (portable, dispersive)	Active ingredients identity by radiation scattering and database matching
NIR spectroscopy (portable, dispersive)	Active ingredients identity by radiation reflection/absorption and database matching
Tests requiring central laboratory	Detects
GC	Quantify volatile organic residual solvents, link to manufacturer
HPLC	Quantify known active ingredients and impurities
NMR spectroscopy	Identify and verify identity of active ingredients, excipients; enhanced structural information
Raman spectroscopy (Fourier transform)	Identify active ingredients, excipients; relative concentrations, coating composition, spatially-resolved information through microscopy
Dissolution tests	Index of bioavailability
Ambient (direct) MS	Screen identity and semiquantitation of active ingredients, excipients, analogs, undeclared compounds, impurities
GC–MS	Confirm identity of volatile ingredients and residual solvents, contaminants, undeclared compounds, and impurities with higher selectivity
LC–MS and tandem MS/MS	Quantify active ingredients, excipients; identify wrong active ingredients with higher selectivity

API = active pharmaceutical ingredient; GC = gas chromatography; HPLC = high-performance liquid chromatography; LC = liquid chromatography; MS = mass spectrometry; NIR = near infrared; NMR = nuclear magnetic resonance.

## References

[1] Nayyar GML, Breman JG, Newton PN, Herrington J (2012). Poor-quality antimalarial drugs in southeast Asia and sub-Saharan Africa. Lancet Infect Dis.

[2] Almuzaini T, Choonara I, Sammons H (2013). Substandard and counterfeit medicines: a systematic review of the literature. BMJ Open.

[3] Mackey TK, Liang BA (2011). The global counterfeit drug trade: patient safety and public health risks. J Pharm Sci.

[4] World Health Organization (2011). Substandard/Spurious/Falsely-labeled/Falsified/Counterfeit Medical Products.

[5] Kwok K, Taylor LS (2012). Analysis of counterfeit Cialis^®^ tablets using Raman microscopy and multivariate curve resolution. J Pharm Biomed Anal.

[6] Lung DD, Gerona RR, Wu AH, Smollin CG (2012). Confirmed glyburide poisoning from ingestion of “street Valium.”. J Emerg Med.

[7] Dorlo TPC, Eggelte TA, de Vries PJ, Beijnen JH (2012). Characterization and identification of suspected counterfeit miltefosine capsules. Analyst (Lond).

[8] Mackey TK, Liang BA, Kubic T, Herrington JE, Nayyar GML, Breman JG (2015). Empirical analysis of counterfeit drug penetration in global legitimate medicine supply chains: a descriptive assessment. Am J Trop Med Hyg.

[9] Campbell N, Clark JP, Stecher VJ, Goldstein I (2012). Internet-ordered Viagra (slidenafil citrate) is rarely genuine. J Sex Med.

[10] Porta M (2014). A Dictionary of Epidemiology.

[11] World Health Organization (2010). New Definition for “Substandard Medicines.”.

[12] Attaran A, Barry D, Basheer S, Bate R, Benton D, Chauvin J, Garrett L, Kickbusch I, Kohler JC, Midha K, Newton PN, Nishtar S, Orhii P, McKee M (2012). How to achieve international action on falsified and substandard medicines. BMJ.

[13] Newton PN, Green MD, Fernández FM (2010). Impact of poor-quality medicines in the ‘developing’ world. Trends Pharmacol.

[14] United States Pharmacopeia (2013). Media Reports on Medicine Quality: Focusing on USAID-Assisted Countries.

[15] PR Newswire (2013). Ten Global Health Organizations United in a Worldwide Campaign to Protect Patients from Fakes.

[16] Institute of Medicine (2013). Countering the Problem of Falsified and Substandard Drugs.

[17] Mackey TK, Liang BA (2013). Improving global health governance to combat counterfeit drugs: a proposal for a UNODC-WHO-Interpol trilateral mechanism. BMC Med.

[18] Newton PN, Lee SJ, Goodman C, Fernández FM, Yeung S, Phanouvong S, Kaur H, Amin AA, Whitty CJM, Kokwaro GO, Lindegårdh N, Lukulay P, White LJ, Day NPJ, Green MD, White NJ (2009). Guidelines for field surveys of the quality of medicines: a proposal. PLoS Med.

[19] World Health Organization (2014). Recommendations on the Content of a Survey Protocol for Surveys of the Quality of Medicines (July 2014). Working document QAS/14.590.

[20] Green MD, Hostetler DM, Nettey H, Swamidoss I, Ranieri N, Fernandez F, Newton PN, Herrington JE, Nayyar GML, Breman JG (2015). Integration of novel low-cost colorometric and visual fluorescent techniques for rapid identification of falsified artemether-lumefantrine and other drugs in resource poor areas. Am J Trop Med Hyg.

[21] United States Pharmacopeia (2009). Survey of the Quality of Selected Antimalarial Medicines Circulating in Madagascar, Senegal, and Uganda.

[22] U.S. Pharmacopeial Convention (2013). PQM in Africa.

[23] Hajjou M, Krech L, Roth L, Pribluda V, El Hadri L, Evans L, Chibwe K, Phanouvong S, Lukulay P, Herrington JE, Nayyar GML, Breman JG (2015). Monitoring the quality of medicines: results from Africa, southeast Asia, and Latin America. Am J Trop Med Hyg.

[24] Ranieri N, Tabernero P, Green MD, Verbois L, Herrington J, Sampson E, Satzger RD, Phonlavong C, Thao K, Newton PN, Witkowski MR (2014). Evaluation of a new handheld instrument for the detection of counterfeit artesunate by visual florescence comparison. Am J Trop Med Hyg.

[25] Food and Drug Administration (2013). FDA Facts: FDA's Counterfeit Detection Device CD-3.

[26] The Indian Express online (2013). India Aims to Clock USD 15.5 Bn Pharma Exports FY13.

[27] Nychis B (2011). Import of Human Drugs and Human Drug Components.

[28] Gostin LO, Sridhar D (2014). Global health and the law. N Engl J Med.

[29] Callard C (2010). Follow the money: how the billions of dollars that flow from smokers in poor nations to companies in rich nations greatly exceed funding for global tobacco control and what might be done about it. Tob Control.

[30] UNICRI (2007). Counterfeiting, A Global Spread, a Global Threat.

[31] Sun X, Xu X, Zhang X (2011). Counterfeit bevacizumab and endophthalmitis. N Engl J Med.

[32] NABP (2013). Internet Drug Outlet Identification Program. Progress Report for State and Federal Regulators.

[33] Tremblay M (2013). Medicines counterfeiting is a complex problem: a review of key challenges across the supply chain. Curr Drug Saf.

[34] Food and Drug Administration (2012). FDA Takes Action Against Thousands of Illegal Internet Pharmacies.

[35] World Health Organization (2003). Effective Medicines Regulation: Ensuring Safety, Efficacy and Quality.

[36] World Health Organization (2004). Availability of Drug Regulatory and Quality Assurance Elements in Member States of the WHO African Region. Brazzaville, Congo.

[37] Ndomondo Sigonda M, Ambali A (2011). The African medicines regulatory harmonization initiative: rationale and benefits. Clin Pharmacol Ther.

[38] World Health Organization (2014). International Medical Products Anti-Counterfeiting Taskforce (IMPACT).

[39] Binagwaho A, Bate R, Gasana NM, Karema C, Mucyo Y, Mwesigye JP, Biziyaremye F, Nutt CT, Wagner CM, Jensen P, Attaran A (2013). Combating substandard and falsified medicines: a view from Rwanda. PLoS Med.

[40] Yeung S, Lawford HLS, Tabernero P, Nguon C, van Wyk A, Malik N, DeSousa M, Rada O, Boravann M, Dwivedi P, Hostetler DM, Swamidoss I, Green MD, Fernandez FM, Kaur H, Herrington JE, Nayyar GML, Breman JG (2015). Quality of antimalarials at the epicenter of antimalarial drug resistance: results from an overt and mystery client survey in Cambodia. Am J Trop Med Hyg.

[41] Kohler JC, Mackey TK, Ovtcharenko N (2014). Why the MDGs need good governance in pharmaceutical systems to promote global health. BMC Public Health.

